# Maintenance of Intraspecific Diversity in Response to Species Competition and Nutrient Fluctuations

**DOI:** 10.3390/microorganisms10010113

**Published:** 2022-01-06

**Authors:** Jorin Hamer, Birte Matthiessen, Silvia Pulina, Giannina S. I. Hattich

**Affiliations:** 1 Marine Ecology, GEOMAR Helmholtz Centre for Ocean Research, 24105 Kiel, Germany; bmatthiessen@geomar.de (B.M.); giannina.hattich@abo.fi (G.S.I.H.); 2 Aquatic Ecology Group, Department of Architecture, Design and Urban Planning, University of Sassari, 07100 Sassari, Italy; pulinasi@uniss.it; 3 Faculty of Science and Engineering, Åbo Akademi University, 20520 Turku, Finland

**Keywords:** intraspecific diversity, nutrient fluctuations, competition, trait variability, phytoplankton, *Emiliania huxleyi*, *Chaetoceros affinis*, genotype coexistence, *V_max_*, cell size

## Abstract

Intraspecific diversity is a substantial part of biodiversity, yet little is known about its maintenance. Understanding mechanisms of intraspecific diversity shifts provides realistic detail about how phytoplankton communities evolve to new environmental conditions, a process especially important in times of climate change. Here, we aimed to identify factors that maintain genotype diversity and link the observed diversity change to measured phytoplankton morpho-functional traits *V_max_* and cell size of the species and genotypes. In an experimental setup, the two phytoplankton species *Emiliania huxleyi* and *Chaetoceros affinis*, each consisting of nine genotypes, were cultivated separately and together under different fluctuation and nutrient regimes. Their genotype composition was assessed after 49 and 91 days, and Shannon’s diversity index was calculated on the genotype level. We found that a higher intraspecific diversity can be maintained in the presence of a competitor, provided it has a substantial proportion to total biovolume. Both fluctuation and nutrient regime showed species-specific effects and especially structured genotype sorting of *C. affinis*. While we could relate species sorting with the measured traits, genotype diversity shifts could only be partly explained. The observed context dependency of genotype maintenance suggests that the evolutionary potential could be better understood, if studied in more natural settings including fluctuations and competition.

## 1. Introduction

Global biodiversity is threatened by five major drivers: habitat change, overexploitation, pollution, introduction of non-indigenous species, and climate change [1,2]. The consequences of biodiversity loss include lower stability of ecosystem functions and a reduced efficiency of communities to take up resources, build up biomass, and recycle nutrients [3]. As a part of biodiversity, genetic or intraspecific diversity has received little attention for a long time [4], although it has been shown to enhance ecosystem recovery [5] and was linked to physiological versatility and ecological resilience towards climatic stress [6]. The ecological effects of intraspecific diversity can be as high or even higher than species effects [7].

Phytoplankton are diverse, globally distributed, and build the base for most marine food webs [8]. They account for approximately half of global primary productivity [9], and are substantial drivers for biogeochemical cycles [10,11]. Phytoplankton diversity is an important factor in structuring marine ecosystems [12]. Climate change-induced sea surface warming results in enhanced stratification, restricting nutrient availability, which is a major driver for phytoplankton composition and biomass [13,14,15]. Future predictions for phytoplankton communities in more stratified open oceans often suspect that smaller phytoplankton will find themselves in positions of advantage due to their ability to effectively take up nutrients at low concentrations [16,17]. However, there is consideration that larger cells could also benefit from the multiple drivers of global change, making predictions about phytoplankton communities in future oceans a difficult task [18]. Predicting diversity changes remains especially difficult, as it not only relies on understanding species coexistence, but also depends on intraspecific diversity.

Since Hutchinson’s “paradox of the plankton”, researchers have been trying to explain how the observed species diversity in a system with limited resources can be maintained [19,20]. While many solutions have been proposed, understanding the underlying mechanisms of species coexistence is still an ongoing topic in community ecology [21,22,23,24]. Interspecific trait variation and associated trade-offs are commonly seen as essential for coexistence, as they represent niche differentiation among species [25,26]. Modern coexistence theory identified a balance between stabilization and differences in species’ competitive abilities, which is required for species to coexist [27,28,29]. Like interspecific trait variation, intraspecific trait variation has been shown to be omnipresent [30,31,32,33]. It can change species interactions and thereby promote or undermine species coexistence [34,35,36,37,38]. While results appear contradictory, it is the type of trait and the trade-off exhibiting intraspecific trait variation that determines the effect on species coexistence [39]. Less is known about the effects of interspecific competition on intraspecific diversity, although the effects of species on genetic diversity have been hypothesized [40].

Moreover, variability of environmental factors has been shown to promote species coexistence by temporal niche partitioning [41,42]. In particular, nutrient fluctuations can prevent competitive exclusion, and as such sustain coexistence of phytoplankton attributable to different nutrient uptake strategies [43,44,45]. Nevertheless, little is known about the effects of nutrient fluctuations on the maintenance of the associated genotype variability, which has been shown experimentally to decline rapidly, while two phytoplankton species stably coexisted at a regularly fluctuating nutrient regime in the long term [46]. Recently it has been found in yeast that nutrient fluctuations maintain higher genetic diversity than static environments [47].

As phytoplankton are largely structured by nutrient availability [21,48,49], traits important for nutrient acquisition and utilization as well as trade-offs between them facilitate the understanding and prediction of community structures [50,51]. Competitive strategies of nutrient acquisition in phytoplankton were proposed by Sommer [43]: affinity-adapted species with low half-saturation constants have an advantage under nutrient limitation, while nutrient repletion favors velocity-adapted species, which have a high maximum nutrient uptake velocity (*V_max_*) and a high maximum growth rate (*µ_max_*). While the use of a half-saturation constant as a measure of nutrient affinity has been heavily criticized and, as an alternative, the use of affinity *α* is recommended [52], the general classification of nutrient strategies still holds and is visible in bloom succession from diatoms to coccolithophores [53,54], typical representatives for the described velocity- and affinity-adapted strategies, respectively. Furthermore, among phytoplankton major taxonomic groups, *V_max_* scales with a two-thirds exponent of cell size based on the relationship of cell surface to cell volume scaling and enzyme kinetics [50]. Cell size has been described as a master trait in phytoplankton because of its scaling with many functional traits [55]. It spans dimensions of 4 to 5 orders of magnitude [56,57] and contributes to the enormous interspecific trait variation described in phytoplankton [50]. Moreover, some studies have also reported substantial intraspecific trait variation in phytoplankton cell size and traits relevant for nutrient acquisition, growth, and toxin production [58,59,60,61]. This large intraspecific trait variation suggests high potential for adaptation, which could compensate for lower fitness of phytoplankton in more adverse conditions [18], and for the same coexistence mechanisms to operate as on species level.

The main objective of this study was to uncover mechanisms maintaining intraspecific diversity in two globally important but fundamentally different marine phytoplankton species. The coccolithophore *Emiliania huxleyi* and the diatom *Chaetoceros affinis* are different in their morpho-functional traits, such as cell shape and size, and nutrient uptake-related traits that are reflected in different nutrient uptake strategies; we assumed that the nine genotypes of each species used in this study also show trait differences. To explain mechanistically the genotype sorting of the two species, we identified the morpho-functional traits cell size and *V_max_* of each genotype individually. With this knowledge, we aimed to assess the effects of interspecific competition, nutrient availability, and temporal fluctuations in nutrient availability on intraspecific diversity. More precisely, we wanted to know (i) whether intraspecific diversity of one species is dependent on the presence of a competing species, (ii) whether intraspecific diversity is dependent on different nutrient concentrations and limitations, and (iii) if additional variability in nutrient availability, represented by changing temporal windows for species or genotypes with either affinity or velocity specialization to thrive, can maintain a higher intraspecific diversity.

## 2. Materials and Methods

### 2.1. Study System and Laboratory Conditions

The populations and communities used in this study comprised species from two major groups of phytoplankton, coccolithophores (*Emiliania huxleyi*) and diatoms (*Chaetoceros affinis*). Both coccolithophores and diatoms are cosmopolitan bloomers, together contributing up to 50% of marine primary production [62,63]. However, their different nutrient-utilization strategies [43,64] are mirrored in the succession of blooms: whereas diatoms initiate and drive the early peak of a bloom by rapid uptake of nutrients followed by rapid growth, coccolithophores occur later in the succession when the nutrient concentration is already lowered and their high affinity is advantageous [53,54]. These different nutrient uptake strategies possibly enable the coexistence of *E. huxleyi* and *C. affinis* in microcosms (Figure S1), which mimic natural shifts in nutrient concentrations from replete to deplete conditions. From each species, we used nine genotypes that were isolated in 2014 and 2015 from waters near Gran Canary (for detail on the genotypes used, see [65]).

The experiments were carried out in 0.5 L polycarbonate bottles (Nalgene) filled with 660 mL sterile filtrated (0.2 µm) artificial seawater (35 salinity; after Kester [66]. The experiment was performed in a climate chamber at 21.9 ± 0.6 °C and settled on a rotating wheel to ensure mixing of their content. Light was supplied by a 17:7 LD cycle (3-h sunrise and sunset) with 299.6 ± 21.0 µmol m^−2^ s^−1^ at maximum light intensity.

### 2.2. Trait Measurements

The maximum uptake rate *V_max_* for nitrogen was determined for each genotype of *Chaetoceros affinis* and *Emiliania huxleyi* by applying a gradient of seven levels of nitrate concentrations while keeping phosphate constant (Table 1). The applied nutrient concentrations differed slightly between the species, as they were for example adjusted to avoid co-limitation of the diatom with silicate. Silicate was added in access to both species by applying an N:Si ratio of 4 and 0.6 in *E. huxleyi* and *C. affinis,* respectively. Each treatment combination of genotype with nitrate concentration was three-fold replicated, which resulted in 189 experimental units per species. Due to space limitation on the plankton wheel, the *V_max_* experiments for *C. affinis* and *E. huxleyi* took place at different times (October 2020 and March 2019, respectively).

In order to ensure maximal uptake, we conducted the experiment with genotypes starting at minimum nitrogen cell quotas (*Q_min_*), i.e., with cells that were starved prior to the experimental onset. Nutrient concentrations in this acclimation batch cycle were 1.8 µmol L^−1^ P, 30 µmol L^−1^ N, and 40 µmol L^−1^ Si for *C. affinis*, and 1.5 µmol L^−1^ P, 15 µmol L^−1^ N, and 3.75 µmol L^−1^ Si for *E. huxleyi*. The acclimation batch cycle lasted 17 and 14 days in *E. huxleyi* and *C. affinis*, respectively. To account for the substantial difference in size between the species, starting volumes were adjusted. All genotypes of *C. affinis* were inoculated with 250 cells mL^−1^, while *E. huxleyi* genotypes started with 1000 cells mL^−1^. Throughout the 4 days of experiment, the individual growth of each culture was followed daily by cell counts and inorganic dissolved nutrients measurements. To quantify *E. huxleyi* abundance, a 1 mL sample was taken and analyzed with a flow cytometer (Beckman Coulter Gallios). The abundance of *C. affinis* was analyzed from 5 mL Lugol’s iodine solution fixed samples under inverted microscopes Axiovert 200 and Axio Observer A1 (Zeiss) in Utermöhl chambers. Inorganic dissolved nutrient samples were taken by filtering a 6 mL sample over prewashed 0.2 µm GF/F filters (Whatman) and analyzed using an autoanalyzer (Thermo Scientific Flash).

Specific uptake (*V*) for each genotype at a given nutrient concentration was determined by choosing the highest per capita nitrate uptake during the experiment. Nitrate uptake was measured as the loss of dissolved nitrate within one day by calculating the difference between nitrate concentrations measured at consecutive days, normalized by the mean number of cells present at those days. The highest per capita uptake rate took place in most replicates between experiment day one and experiment day two. To calculate *V_max_* of each genotype, the determined specific uptake rates were fitted to a Monod model by using the starting nitrate concentrations.

The size of each genotype was calculated after Hillebrand [67] from the diameter/ width and length measurements. To account for high variability in size within single *C. affinis* genotypes, we quantified the proportion of different size classes and measured the sizes of five cells per class and replicate. For *E. huxleyi* starvation prior the start of the trait measurements might result in considerably different cell sizes [68]. Consequently, the sizes of 15 cells per replicate were assessed at the start of the diversity experiment to facilitate a comparison of single genotype traits to resulting diversity shifts.

### 2.3. Diversity Experiment

#### 2.3.1. Experimental Design and Setup

**Figure 1:**
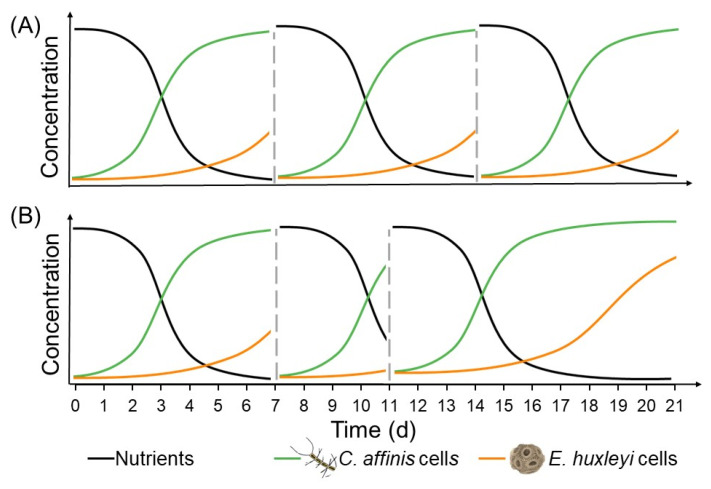
Schematic view of nutrient availability under (**A**) regular (transfer every 7 days; dashed grey lines indicate time of transfer) and (**B**) irregular (transfer after 7, 4 and 10 days; dashed grey lines) fluctuations and consequently changing temporal windows for species to thrive.

The effects of the presence of a competitor and fluctuations in nutrient availability on genotypic diversity were tested under different nutrient regimes using an experimental setup with the two phytoplankton species *Emiliania huxleyi* and *Chaetoceros affinis.* The two species were cultivated separately in mono-cultures and together in mix-cultures using a semi-continuous batch cycle system, where part of the community was transferred into new media to mark the start of a new batch cycle. To achieve the intended different nutrient fluctuations (regular versus irregular), the lengths of the batch cycles were varied (Figure 1). For half of the bottles, a part of the cells was transferred into new bottles with fresh media every 7 days (regular fluctuations at fixed batch cycle length), while for the other half a part of the cells was transferred after 7, 4, or 10 days (irregular fluctuations at variable batch cycle length), in a recurring fashion. Three different nutrient regimes were applied to reflect different N:P ratios and limitation scenarios in the ocean. Across these nutrient regimes, phosphate concentration was held equal with 0.93 ± 0.09 µmol L^−1^, while nitrate levels were adjusted to mimic a 10N:1P, 20N:1P, and 30N:1P ratio, and reached final nitrate concentrations of 8.60 ± 0.62, 19.08 ± 0.32, or 29.36 ± 0.47 µmol L^−1^, respectively. Silicate concentrations were aligned to reflect a 4:1 N:Si ratio. This relatively low N:Si ratio was chosen despite the potential of *C. affinis* to get co-limited by silicate, as preliminary tests of different nutrient concentrations demonstrated that this allowed for the longest coexistence of the model species in laboratory settings. Selenium, vitamins, and trace metals were also held constant according to f/8 concentration [69]. The culture treatment (mono-culture and mix-culture) was fully crossed with the batch cycle length treatment (fixed and variable) and the nutrient regimes (10N:1P, 20N:1P and 30N:1P), and replicated five times, resulting in 90 experimental units.

Prior to the experimental start, the genotypes were separately acclimated to experimental conditions with 20 µmol L^−1^ nitrate for 7 days. In the mix-culture, species were inoculated in concentrations of 25 cells mL^−1^ (*C. affinis*) and 500 cells mL^−1^ (*E. huxleyi*) to reflect similar biovolumes. The nine different genotypes of each species contributed equally to the total concentrations of the species. Mono-cultures were inoculated with 50 cells mL^−1^ (*C. affinis*) or 1000 cells mL^−1^ (*E. huxleyi*). The experiment ran for 91 days, or 13 batch cycles in total.

#### 2.3.2. Sampling and Transfer

At the end of each batch cycle, bottles were sampled and transferred to the subsequent batch cycle under a biosafety cabinet (NuAire, model: NU-480-400E). For measurements of *E. huxleyi* density by a Gallios flow cytometer (Beckman Coulter), 3 mL volume was sampled over a 20 µm mesh-size sieve, which separated *E. huxleyi* cells from the significantly larger *C. affinis.* Another 5 mL were fixed with Lugol’s iodine solution for a cell count of *C. affinis*, and the cell size of both species was measured under inverted microscopes, as described above.

Part of the volume from the bottles with cells was transferred into new bottles with fresh media, marking the start of a new batch cycle. At first, transfer volumes for each culture and batch cycle length combination across all nutrient regimes were calculated, with the information on *E. huxleyi* density and one counted replicate each of *C. affinis*, to ensure a minimum initial density of 10 cells mL^−1^ of each species. With the start of the fifth batch cycle, the method was adjusted; transfer volumes were calculated individually for each nutrient treatment, and in the *E. huxleyi* mono-cultures for each bottle, to ensure that the starting densities in all treatments were comparable.

To follow the individual growth curve of each species during a batch cycle, in addition to the weekly sampling, the first long batch cycle (i.e., the third batch cycle) was characterized by daily measurements of cell density (*E. huxleyi*) and fluorescence (*C. affinis*) (Turner Designs, model: 10-AU Fluorometer). Nitrate, silicate, and phosphorus concentrations were measured at the end of the first three batch cycles in the mix-cultures at variable batch cycle length, to verify the proposed effects on nutrient concentrations at batch cycles of different lengths (Figure 1). Furthermore, nutrient concentrations in mix-cultures at the seventh batch cycle were measured daily from day 4 to day 7, to see which nutrient was the first to be completely taken up in each nutrient regime. The nutrient measurements were carried out in the same way as that described for the trait measurements.

#### 2.3.3. Genotype Distribution

At midterm (i.e., 49 days or the seventh batch cycle) and at the end of the experiment (i.e., 91 days or the thirteenth batch cycle), subsamples were taken from each bottle to reisolate *E. huxleyi* and *C. affinis* cells for assessment of the genotype distribution, using microsatellites (after Hattich [65] and Listmann [46]; see the Supplementary Materials for details). Sequencing data were analyzed using GeneMarker software. Isolate genotypes were identified by comparing the primer peaks of the reisolates with the primer peaks of the stem culture genotypes. Experimental units with less than 5 isolates of one species identified were excluded from further analyses.

#### 2.3.4. Shannon’s Diversity Index

Shannon’s diversity index H’ [70] was adapted on the genotype level and calculated separately for each species in every experimental unit, using the information on genotypes’ relative abundance. Calculation was carried out for both species separately to disentangle whether maintenance of intraspecific diversity varies between the species. The calculation (1) requires information about the number of genotypes S present, as well as their proportions p_i_, making Shannon’s diversity index H’ a comprehensive measure of diversity:

(1) $$H^{\prime }=\;-\overset{S}{\underset{i=1}{\sum }}\;p_{i}\ln p_{i\;}$$

#### 2.3.5. Statistical Analysis

Shannon’s diversity index and the relative abundances of the most frequent genotypes *C91*, *C41*, *B82*, *B57*, and *B67* were analyzed by using mixed-effects models with four fixed factors: batch cycle length, nutrient regime, culture, and time. Bottle identity was incorporated as a random factor to account for repeated measures. Assumptions of tests were validated graphically, and the significance level for all analyses was set to *p* < 0.05. Starting from the most complex model (with all possible interactions), careful model simplification was applied. Model selection followed biological reasoning and the Bayesian information criterion (BIC). Final model output was reported as type II Wald F tests using Kenward-Roger df. Where test assumptions were not met, generalized linear mixed-effects models were used and reported as type II Wald χ^2^ tests.

All statistical analyses were done using R software [71] and additional packages lme4 [72], car [73], plyr [74], and ggplot2 [75].

## 3. Results

### 3.1. Trait Variability

**Figure 2:**
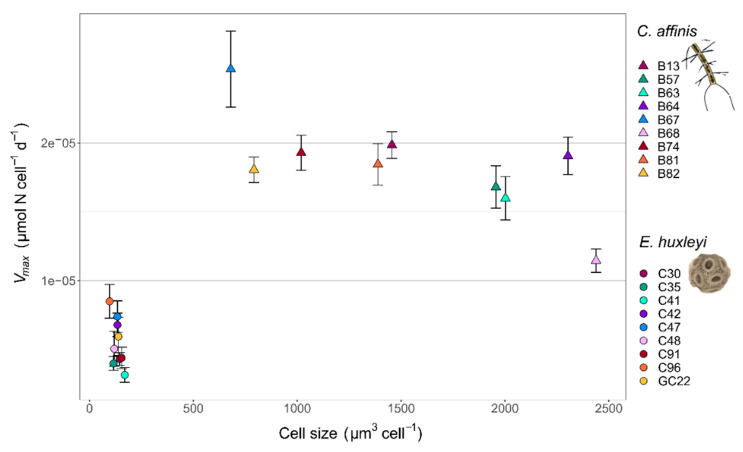
*V_max_* and cell size of the nine genotypes for *E. huxleyi* and *C. affinis* respectively. Error bars of *V_max_* show SE of Monod model estimates.

Trait measurements of *Chaetoceros affinis* and *Emiliania huxleyi* genotypes revealed fundamental trait differences between the two species and among the genotypes of each species, with regard to cell size and the maximum nutrient uptake rate *V_max_* (Figure 2). Between species, the generally smaller *E. huxleyi* genotypes had a mean cell size of 135 ± 20 µm^3^ in relation to the mean cell size of 1560 ± 612 µm^3^ for the larger *C. affinis* genotypes. In correlation with cell size, the mean *V_max_* was higher in *C. affinis* (1.8 × 10^−5^ ± 3.5 × 10^−6^ µmol N cell^−1^ d^−1^) than in *E. huxleyi* (5.5 × 10^−6^ ± 1.7 × 10^−6^ µmol N cell^−1^ d^−1^), showing the differences in nutrient uptake strategies of the species. In comparison, among the genotypes of each species, there was no correlation of *V_max_* with size. Intraspecific trait variability in *E. huxleyi* was reflected in a cell size range of 97 µm^3^ to 170 µm^3^ and a *V_max_* range of 3.1 × 10^−6^ µmol N cell^−1^ d^−1^ to 8.5 × 10^−6^ µmol N cell^−1^ d^−1^ in the genotypes. In the *C. affinis* genotypes, the sizes ranged from 680 µm^3^ to 2439 µm^3^ and *V_max_* ranged from 1.1 × 10^−5^ µmol N cell^−1^ d^−1^ to 2.5 × 10^−5^ µmol N cell^−1^ d^−1^. Standardized to the species mean, *C. affinis* showed higher variability in size (from −56% to 56%) compared to *E. huxleyi* (from −28% to 26%), whereas the variability in *V_max_* was higher in *E. huxleyi* (from −43% to 55%) than in *C. affinis* (from −37% to 39%).

### 3.2. Diversity Changes

#### 3.2.1. Genotype Sorting

**Figure 3:**
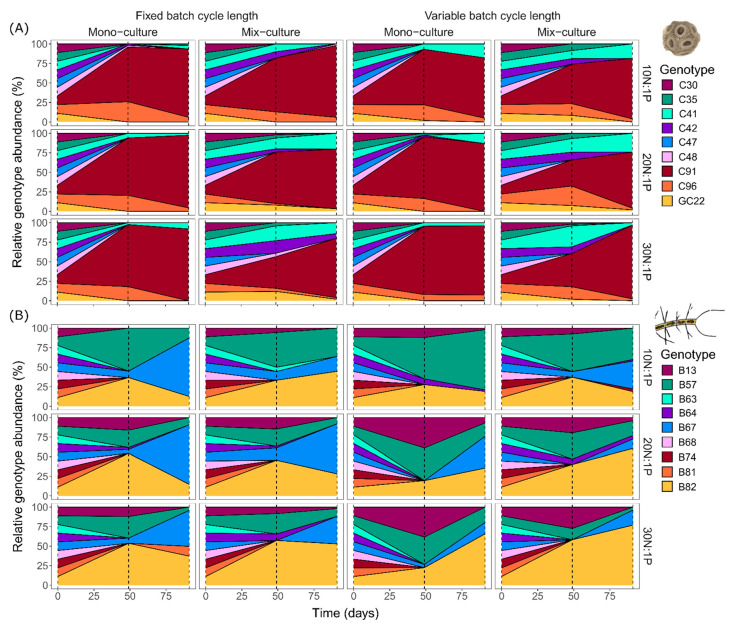
Mean relative genotype composition of *E. huxleyi* (**A**) and *C. affinis* (**B**) genotypes over time in mono-culture and mix-culture, at three nutrient regimes (10N:1P, 20N:1P, and 30N:1P) with fixed and variable batch cycle length.

**Figure 4:**
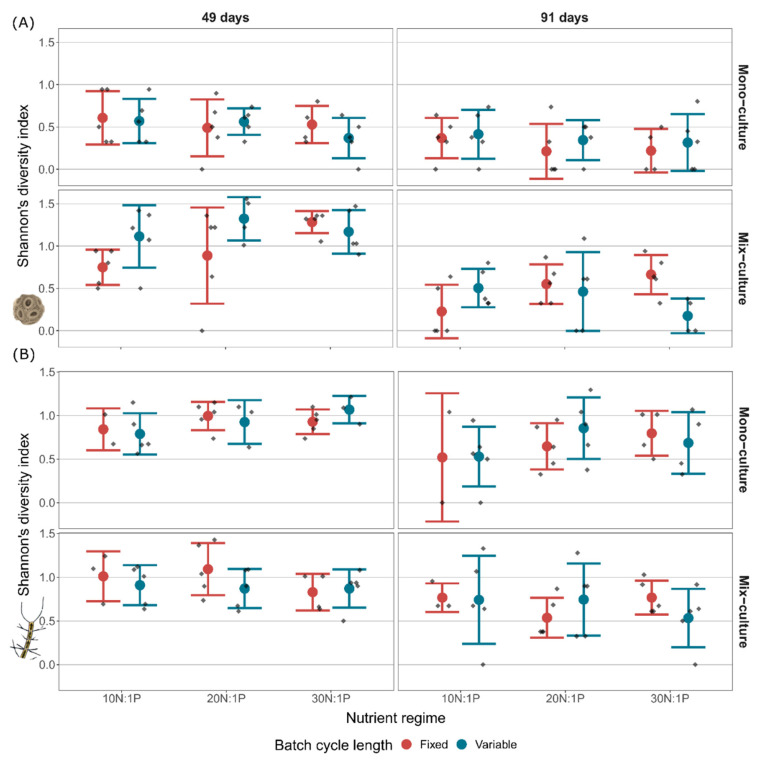
Shannon’s diversity of *E. huxleyi* (**A**) and *C. affinis* (**B**) genotypes at midterm (i.e., after 49 days) and at the end of the experiment (i.e., at 91 days) in mono-culture and mix-culture, at three nutrient regimes (10N:1P, 20N:1P, and 30N:1P) with fixed and variable batch cycle length. Mean ± SD and data points are shown.

Genotype sorting of both *E. huxleyi* and *C. affinis* changed fundamentally over time (Figure 3), and the dynamics differed significantly between the two species. In *E. huxleyi* at midterm of the experiment, across all treatments, eight of the original nine genotypes were found. Their relative contributions to *E. huxleyi* total abundance changed drastically compared to the initial equal distribution, and led to dominance of genotype *C91* in all treatments. Despite this uniform dominance, differences among treatments occurred in the maintenance of genotypes over time. The most prominent change is reflected in two more remaining genotypes in mix-cultures compared to mono-cultures. At the end of the experiment, the remaining genotypes were further reduced to six and the dominance of genotype *C91* across all treatments increased even more, to nearly monodominance. In contrast to *E. huxleyi*, four genotypes majorly contributed to total *C. affinis* abundance throughout the experiment and dominated the different treatments in distinct ways. In addition, the two species differed in the exclusion process of genotypes; in *C. affinis*, this process was initially accelerated but in the longer term slower. More specifically, at midterm, a total of only six of the original nine genotypes were found, while at the end a total of seven genotypes were still present. Furthermore, the dominance of the single genotypes was not as pronounced as that described for *E. huxleyi*. This divergence in genotype sorting and exclusion between species is reflected in a 35% higher Shannon’s diversity index in *C. affinis* compared to *E. huxleyi* (Figure 4).

#### 3.2.2. Maintenance of Intraspecific Diversity

##### Emiliania Huxleyi

In all treatments, the described change in genotype-sorting was reflected in a significant decrease of Shannon’s diversity index of *E. huxleyi* genotypes over time (Figure 4; “Time”, Table 2). The loss of Shannon’s diversity was, however, not uniform across treatments, but affected by culture, batch cycle length, and nutrient regime. Culture was especially important for the maintenance of Shannon’s diversity, as it was not only involved in several interactions, but mix-cultures generally increased diversity (“Culture”, Table 2), reflected in a 107% higher Shannon’s diversity in the mix-culture compared to mono-cultures at midterm. Coinciding with the higher Shannon’s diversity in mix-cultures, a reduction of the dominant genotype *C91* (“Culture”, Table 3) and an increase of a subdominant genotype *C41* (“Culture”, Table 4) were observed. Additionally, with rising nitrate concentration, Shannon’s diversity increased in mix-cultures but not in mono-cultures (“Culture × Nutrient”, Table 2). This increase in diversity correlated, once again, with a decrease in the proportion of genotype *C91* (“Culture × Nutrient”, Table 3). Furthermore, batch cycle length affected Shannon’s diversity index through interactions (“Batch cycle length × Nutrient” and “Batch cycle length × Nutrient × Culture”, Table 2). More specifically, variable batch cycle length had no visible effect on diversity in mono-cultures at the end of the experiment, but led to 121% higher diversity compared to the fixed batch cycle length in the mix-cultures at the low nutrient regime 10N:1P. In contrast, variable batch cycle length decreased Shannon’s diversity index by 74% compared to the fixed batch cycle length at the high nutrient regime 30N:1P in the mix-cultures.

##### Chaetoceros Affinis

Shannon’s diversity index of *C. affinis* genotypes significantly decreased with time as a result of increased dominance of the four major contributing genotypes (Figure 4; “Time”, Table 5). Diversity was interactively affected by culture and nutrient regime (“Culture × Nutrient”, Table 5). This interaction was reflected in a 12% diversity decline from 10N:1P to 30N:1P in the mix-cultures compared to a 29% increase with the nutrient regime in the mono-cultures. The composition of *C. affinis* genotypes was largely structured by nutrients. The 20N:1P and 30N:1P nutrient regimes were dominated by genotype *B82* (“Nutrient”, Table 6), while the 10N:1P regime was mainly composed of genotype *B57* (“Nutrient”, Table 7). Variable batch cycle length led to a higher proportion of genotype *B57* (“Batch cycle length”, Table 7), while fixed batch cycle length favored genotype *B67* (“Batch cycle length”, Table 8). Additionally, genotype *B67*, which at midterm in some of the samples was below detection limit, increased with time in all treatments (“Time”, Table 8) and genotype *B82* was more abundant in mix-cultures compared to mono-cultures (“Culture”, Table 6).

## 4. Discussion

Trait measurements of *Emiliania huxleyi* and *Chaetoceros affinis* genotypes indicated inter- and intraspecific trait variability with respect to size and the maximum nitrate uptake rate, *V_max_*. While *E. huxleyi* showed a higher percentage of variability in *V_max_*, *C. affinis* exhibited higher variability in size. The results of the diversity experiment showed that while genotype sorting of both species changed considerably over time, the patterns of the dynamics differed between species. As such, the genotype diversity of the two species was altered in different ways by the experimental treatments culture, batch cycle length, and nutrient regime. Shannon’s diversity in *E. huxleyi* was maintained by interspecific competition through the presence of *C. affinis*, and by irregular fluctuations in the 10N:1P nutrient regime, whereas *C. affinis* diversity was maintained by interspecific competition in the 10N:1P nutrient regime.

### 4.1. Maintenance of Intraspecific Diversity

#### 4.1.1. Effects of Interspecific Competition

Our results showed that intraspecific diversity of one species can be affected by the presence of another and indicated that the coexistence of species might play an important role in the maintenance of intraspecific diversity. Interspecific competition could change intraspecific competition, resulting in higher intraspecific diversity. Specifically, the Shannon’s diversity of *E. huxleyi* was maintained for longer when cultivated together with *C. affinis* in a mix-culture compared to cultivation in a mono-culture. One possible explanation could be that the co-occurring growth of *C. affinis* effectively changed the nutrient concentrations or ratios, and by this process altered the competition between the *E. huxleyi* genotypes. As indicated by its higher cell size and *V_max_*, *C. affinis* (when compared to *E. huxleyi*) represents the better competitor for nutrients under replete nitrate conditions, and as such it likely altered the nutrient regime at the beginning of a batch cycle towards limiting to depleted conditions. In contrast to *C. affinis*, *E. huxleyi* is an affinity-adapted species, reflected in its smaller size and lower *V_max_*. As such, the rapid depletion of nutrients by the diatom at the onset of a batch cycle likely favored certain *E. huxleyi* genotypes and led to the decelerated exclusion speed of *E. huxleyi* genotypes. This could be regarded as an example of the facilitation described by Vellend, as shown in models to increase genotype richness as a result of higher species richness [76]. The observation also agrees with certain findings in a plant community, where maintenance of genetic variation was reported to depend on species diversity [77].

In *C. affinis*, the presence of the competitor altered Shannon’s diversity depending on nutrient conditions. Our results suggested that the effect of the interspecific competitor on diversity was mediated by species evenness, which in turn was driven by the nutrient regime (Figure S1). Evenness and as such the contribution of *E. huxleyi* to the total biovolume were highest in the 10N:1P nutrient regime. Only in the 10N:1P nutrient regime a positive effect of the presence of *E. huxleyi* could be measured. In the 30N:1P nutrient regime, the *E. huxleyi* biovolume contribution was negligible, and the influence of interspecific competition on the intraspecific diversity of *C. affinis* was highly unlikely. This suggests that the promotion of intraspecific diversity of a species requires substantial contribution of the competitor species to total biomass.

Furthermore, our results showed that genotype identity, which underlies intraspecific diversity, was altered by the presence of a competitor in both *E. huxleyi* and *C. affinis.* This was reflected, for example, by the higher relative abundances of genotypes *C41* and *B82* in mix-cultures compared to mono-cultures, respectively. Relative abundance shifts might have resulted from trait variability among genotypes, allowing for diverging responses to trait space shifts driven by interspecific competition. For example, in *E. huxleyi* mono-cultures, the higher *V_max_* of genotype *C91* compared to *C41* could have been beneficial. However, in the presence of *C. affinis*, with a much higher *V_max_* than *E. huxleyi*, nutrient availability for *E. huxleyi* likely decreased and therefore genotype *C41* gained in terms of relative contribution. While it is known that intraspecific trait variation can change species’ interactions [34,35], our results underscore the hypothesized effects of species on genetic diversity [40].

#### 4.1.2. Effects of Nutrient Fluctuations

As nutrient fluctuations can sustain species coexistence [43], we assumed that mechanisms of coexistence are the same on the genotype level and, therefore, additional variability promotes intraspecific diversity. Contrary to our expectations, the outcome from our work showed no generally higher intraspecific diversity when applying irregular rather than regular nutrient fluctuations, in the form of batch cycle length that changed nutrient availability (Figure S3). This outcome was especially surprising, considering that in yeast, nutrient fluctuations maintain higher genetic diversity than static environments [47]. These contradictory findings were likely to have been dependent on the different setups of fluctuations. First, with our fluctuation treatment, the quality and quantity of nutrients supplied was not altered. Second, in contrast to the cited studies that were conducted in chemostats [43,47], in our approach nutrient concentrations were allowed to shift from replete to deplete conditions in both fluctuation environments (Figure S4). Consequently, we did not apply a constant, static environment, but rather mimicked more natural conditions in our system, such as the occurrence and fluctuations in mixing events, which apparently resulted in a low effect of fluctuations on diversity.

Nevertheless, we found that for *E. huxleyi* only in the 10N:1P nutrient regime, variable batch cycle length led to higher Shannon’s diversity than did the fixed batch cycle length. As nitrate was the first nutrient to be depleted in the 10N:1P regime (Figure S4), this suggests that in our system, nitrate limitation was needed for irregular nutrient fluctuations to be of advantage. With respect to species level, it has been shown that nutrient fluctuations can increase macroalgae diversity in nutrient-limited coastal communities, while suppressing diversity in nutrient-enriched communities [78]. The authors explained their finding with the hump-shaped relationship between productivity and diversity [79], through which enrichment resulted in a diversity gain at low productivity sides and a loss in high productivity sides [80,81]. At first sight, our data verified this pattern at the genotypic level, and explained both the observed increase in genotype variation in *E. huxleyi* in the 10N:1P nutrient regime and the observed decline in genotype variation in the 20N:1P and 30N:1P nutrient regimes. Based on stoichiometry and confirmed by nutrient measurements (Figure S3), the 10N:1P regime represents a deplete regime and the 20N:1P and 30N:1P represent replete regimes, in terms of nitrate. However, considering phosphate levels, the 30N:1P regime would be a deplete regime, although the irregular fluctuations suppressed intraspecific diversity rather than promote it. As our fluctuating nutrients did not lead to enrichment but supplied the same amount of nutrients at different growth stages, it is likely that we did not observe a direct effect of a productivity-diversity relationship. However, an indirect effect in the mix-culture was possible, as the relative share of *E. huxleyi* in mix-cultures was significantly higher at 10N:1P than at 30N:1P (Figure S1), which might have prevented rare genotypes from being excluded. At 30N:1P, the very low abundance of *E. huxleyi* could have resulted in an increased likelihood of stochastic extinctions of genotypes, which was intensified by irregular fluctuations, presumably in the short batch cycles where the lowest *E. huxleyi* contribution occurred.

In our experiment, fluctuations did not affect the Shannon’s diversity of *C. affinis*, but did affect the remaining genotype identity. The fact that Shannon’s diversity was not affected by fluctuations could be due to the generally higher genotype diversity of *C. affinis* compared to *E. huxleyi*; in *C. affinis*, this resulted in a similar dominance pattern among treatments, however, with different genotypes. As such, Shannon’s diversity did not capture the observed genotype diversity shifts of *C. affinis*. Another potential explanation is that short, normal, and long batch cycles did not constitute a regulating force on the Shannon’s diversity of *C. affinis* because they all captured the end of growth or stationary phase that is reached on the fifth day (Figure S2). Furthermore, the long batch cycle of the irregular fluctuations did not impose a selection pressure on *C. affinis* as, contrary to our expectation, the long stationary phase did not result in senescence of *C. affinis* (Figure S1).

Irregular fluctuations promoted genotype *B57*, whereas regular fluctuations benefited genotype *B67*. As genotype *B67* showed the highest *V_max_* of all genotypes, this could mean that the regular fluctuations favored genotypes that gained competitive advantage through a high *V_max_*. In contrast, the importance of being a good competitor due to high *V_max_* seemed lower at irregular fluctuations as the proportion of genotype *B57* with a lower *V_max_* increased. This could be driven by the long batch cycle, where other traits such as affinity or storage capacity might have been more important. This shows that even small environmental differences resulting from irregular versus regular fluctuations can select for different genotypes that are specialized to a specific environment.

#### 4.1.3. Effects of Nutrients

The role of nutrients on Shannon’s diversity remained concealed in interactions with the other treatments, as discussed. However, and specifically in *C. affinis*, genotype identity was structured by the nutrient regime. The considerably stronger effect of nutrient regimes on the genotype sorting of *C. affinis* compared to *E. huxleyi* was surprising, considering that (i) *C. affinis* was potentially co-limited by silicate across all nutrient regimes (Figure S4), and (ii) the *C. affinis* percentage trait variability in *V_max_* was smaller than that of *E. huxleyi*. Similar patterns were observed in a previous study, where the genotype sorting of *C. affinis* was more affected by CO_2_ concentration than was the genotype sorting of *E. huxleyi* (after 64 days; [46]), although the response variability in growth rate changes to CO_2_ was lower in *C. affinis* [65]. The authors discussed the overriding effects of general laboratory conditions on *E. huxleyi* genotype sorting, which likely also explain the dominance of the same *E. huxleyi* genotype across all treatments in this study. Only the relative abundance of *E. huxleyi* genotype *C91* decreased with rising nitrate in the presence of *C. affinis*, thereby driving the according change in Shannon’s diversity. However, as this effect was only observable in competition with *C. affinis*, and the biomass of *C. affinis* increased with rising nitrate concentration (Figure S1), this indicates that *C. affinis* had a stronger effect on nutrient availability for *E. huxleyi* genotypes than did the nutrients provided at the start of a batch cycle.

Genotype sorting of *C. affinis* showed that the 20N:1P and 30N:1P nutrient regimes were dominated by genotype *B82*, while the 10N:1P regime was dominated by genotype *B57*. It is well known that higher *V_max_* found in diatoms are advantageous under high nutrient environments in relation to fundamental relationships such as cell surface to cell volume scaling and enzyme kinetics [50]. Trait measurements revealed that genotype *B82* had a slightly higher *V_max_* than *B57*, indicating that a higher *V_max_* could be beneficial at higher nitrate concentrations. However, differences in V_max_ between these *C. affinis* genotypes were small, and thus not sufficient to explain the genotype sorting. Additional trait measurements of *V_max_* for phosphate and silicate could have provided useful information, especially as they might also have been limiting. This applies particularly to potential phosphate limitation in the 30N:1P regime and silicate for *C. affinis* across treatments (Figure S4), potentially restricting the explanatory power of *V_max_* for nitrate. Cell size trait measurements do not explain genotype sorting at the different nutrient regimes, which could be caused by the absence of a correlation with *V_max_* among the genotypes. *V_max_* typically scales with phytoplankton cell size [64,82]; however, we found this relationship only between species. Similar patterns have been found for trade-offs between species that did not operate within species [39,83,84]. Moreover, the limited potential of trait measurements to explain genotype sorting is likely caused by the difference between the fundamental niche in absence of genotype and species competition and the realized ecological niche expressed under competition [85]. A quantitative study on the more than 60 year old concept supported the hypothesis that the fundamental niche is larger than the realized niche, and thereby underlines the importance of biotic interactions for species survival [86]. As genotypes show trait variability as well, this concept should also apply on the intraspecific level, and therefore lead to differences between fundamental and realized niches, restricting the explanatory power of individual trait measurements.

### 4.2. Implications for Phytoplankton in Future Oceans

Understanding the mechanisms by which genotypes coexist is an important step in grasping how biodiversity is maintained in nature. It provides realistic detail about the capability of communities to respond to new environments, a process especially important in times of global change. Our findings show that maintenance of intraspecific diversity is context-dependent, suggesting that the potential for species and communities to cope with altered conditions could be better understood, if it was studied in more natural settings including fluctuations and competition. In particular, the presence of a competitor showed pronounced direct effects on intraspecific diversity, in addition to the indirect modulation effects of other drivers. The competition effects were species-specific and depended on species composition, underlining the importance of assessing evolutionary change in response to new environmental conditions in an ecological context. Disentangling and understanding evolutionary change, and thus genotype sorting, in phytoplankton communities will help in assessing the potential for phytoplankton to cope with climate change, and ultimately improve predictions about their future. These predictions are essential, as shifts in phytoplankton can have cascading effects on ecosystems, which can eventually affect their services provided to humans [87].

## Figures and Tables

**Table 1:** Nutrient levels applied to measure uptake rates *V* at different concentrations of nitrogen for each genotype of *E. huxleyi* and *C. affinis*.

*E. huxleyi*				*C. affinis*			
N:P	Nitrate (μmol L^1^)	Phosphate (μmol L^−1^)	Silicate (μmol L^−1^)	N:P	Nitrate (μmol L^−1^)	Phosphate (μmol L^−1^)	Silicate (μmol L^−1^)
1.7	2.5	1.5	0.625	1.25	2.5	2	3.75
3.3	5	1.5	1.25	2.5	5	2	7.5
0.66	7.5	1.5	1.875	3.75	7.5	2	11.25
6.7	10	1.5	2.5	6.25	12.5	2	18.75
10	15	1.5	3.75	10	20	2	30
13.3	20	1.5	5	15	30	2	45
20	25	1.5	6.25	20	40	2	60

**Table 2:** Analysis of deviance table of Type II Wald F tests with Kenward-Roger df for linear mixed-effects model with Shannon’s diversity index of *E. huxleyi* as response variable. Stars show significance level of the test; *** *p* < 0.001 and * *p* < 0.05.

	F	Df	Df.res	Pr (>F)
Time	84.7822	1	52.640	1.481 × 10^−12^ ***
Culture	32.5963	1	50.472	6.007 × 10^−7^ ***
Batch cycle length	0.7750	1	50.487	0.38284
Nutrient	0.1759	1	49.870	0.67670
Time × Culture	22.7148	1	52.654	1.521 × 10^−5^ ***
Time × Batch cycle length	0.9863	1	52.706	0.32520
Time × Nutrient	0.8258	1	52.042	0.36767
Culture × Batch cycle length	0.2098	1	50.460	0.64893
Culture × Nutrient	4.4990	1	49.904	0.03890 *
Batch cycle length × Nutrient	4.6540	1	49.904	0.03582 *
Time × Culture × Batch cycle length	6.0934	1	52.750	0.01684 *
Culture × Batch cycle length × Nutrient	3.6805	1	49.879	0.06078

**Table 3:** Analysis of deviance table of Type II Wald F tests with Kenward-Roger df for linear mixed-effects model using the proportion of *E. huxleyi* genotype *C91* as response variable. Stars show significance level of the test; *** *p* < 0.001 and * *p* < 0.05.

	F	Df	Df.res	Pr (>F)
Time	58.1324	1	51.725	5.175 × 10^−10^ ***
Culture	23.7114	1	51.418	1.108 × 10^−5^ ***
Batch cycle length	2.1223	1	51.440	0.1512479
Nutrient	0.0035	1	50.787	0.9531272
Time × Culture	13.0523	1	51.737	0.0006851 ***
Time × Batch cycle length	0.1128	1	51.828	0.7383192
Time × Nutrient	1.0086	1	51.123	0.3199753
Culture × Batch cycle length	1.3502	1	51.413	0.2506088
Culture × Nutrient	5.8993	1	50.823	0.0187236 *
Batch cycle length × Nutrient	4.1937	1	50.827	0.0457573 *
Time × Culture × Batch cycle length	6.4147	1	51.821	0.0143857 *
Time × Culture × Nutrient	2.9749	1	51.110	0.0906086

**Table 4:** Analysis of deviance table of Type II Wald χ^2^ tests for generalized linear mixed-effects model using the proportion of *E. huxleyi* genotype *C41* as response variable. Stars show significance level of the test; *** *p* < 0.001 and * *p* < 0.05.

	Chisq	Df	Pr (>Chisq)
Time	0.0585	1	0.80881
Culture	15.5260	1	8.138 × 10^−5^ ***
Batch cycle length	2.5694	1	0.10895
Nutrient	1.1402	1	0.28562
Time × Culture	4.5431	1	0.03305 *
Time × Batch cycle length	0.1090	1	0.74130
Time × Nutrient	2.3381	1	0.12625
Culture × Batch cycle length	0.0601	1	0.80627
Culture × Nutrient	0.7253	1	0.39441
Batch cycle length × Nutrient	3.4933	1	0.06162
Time × Batch cycle length × Nutrient	5.2336	1	0.02216 *

**Table 5:** Analysis of deviance table of Type II Wald F tests with Kenward-Roger df for linear mixed-effects model with Shannon’s diversity index of *C. affinis* as response variable. Stars show significance level of the test; *** *p* < 0.001 and * *p* < 0.05.

	F	Df	Df.res	Pr (>F)
Time	19.6650	1	52.089	4.793 × 10^−5^ ***
Culture	0.0003	1	48.398	0.98596
Batch cycle length	0.1554	1	48.594	0.69518
Nutrient	0.1890	1	48.903	0.66565
Culture × Nutrient	4.1334	1	48.354	0.04755 *

**Table 6:** Analysis of deviance table of Type II Wald F tests with Kenward-Roger df for linear mixed-effects model using the proportion of *C. affinis* genotype *B82* as response variable. Stars show significance level of the test; *** *p* < 0.001 and ** *p* < 0.01.

	F	Df	Df.res	Pr(>F)
Time	1.7182	1	51.616	0.1957293
Culture	7.2690	1	49.769	0.0095460 **
Batch cycle length	0.0185	1	49.688	0.8924912
Nutrient	21.7629	1	49.853	2.348 × 10^−5^ ***
Time × Culture	0.7028	1	51.632	0.4057090
Time × Batch cycle length	12.2608	1	51.615	0.0009635 ***
Time × Nutrient	3.0196	1	51.781	0.0882072
Culture × Batch cycle length	0.1154	1	49.722	0.7355318
Culture × Nutrient	0.2061	1	50.039	0.6518140
Batch cycle length × Nutrient	2.8502	1	49.965	0.0975929
Time × Batch cycle length × Nutrient	3.5864	1	51.788	0.0638450

**Table 7:** Analysis of deviance table of Type II Wald F tests with Kenward-Roger df for linear mixed-effects model using the proportion of *C. affinis* genotype *B57* as response variable. Stars show significance level of the test; *** *p* < 0.001 and ** *p* < 0.01.

	F	Df	Df.res	Pr(>F)
Time	12.5416	1	52.554	0.0008436 ***
Culture	2.8976	1	50.824	0.0948221
Batch cycle length	9.7939	1	50.813	0.0028994 **
Nutrient	30.8335	1	50.911	1.025 × 10^−6^ ***
Time × Culture	0.0809	1	52.644	0.7771768
Time × Batch cycle length	2.8282	1	52.610	0.0985496
Culture × Batch cycle length	1.6830	1	50.853	0.2003867
Batch cycle length × Nutrient	8.1682	1	50.957	0.0061598 **
Time × Culture × Batch cycle length	2.9434	1	52.680	0.0921079

**Table 8:** Analysis of deviance table of Type II Wald χ^2^ tests for generalized linear mixed-effects model using the proportion of *C. affinis* genotype *B67* as response variable. Stars show significance level of the test; *** *p* < 0.001.

	Chisq	Df	Pr(>Chisq)
Time	94.3394	1	<2.2 × 10^−16^ ***
Culture	1.2037	1	0.2725788
Batch cycle length	13.1676	1	0.0002848 ***
Nutrient	0.0439	1	0.8340921
Time × Culture	2.5019	1	0.1137088
Time × Batch cycle length	0.0032	1	0.9545872
Time × Nutrient	1.1567	1	0.2821593
Culture × Batch cycle length	1.5540	1	0.2125526
Culture × Nutrient	0.6527	1	0.4191354
Batch cycle length × Nutrient	0.3337	1	0.5634860
Culture × Batch cycle length × Nutrient	2.9239	1	0.0872775

## Data Availability

Data are available at PANGAEA (www.pangaea.de).

## Supplementary Materials

The following materials are available online at https://www.mdpi.com/article/10.3390/microorganisms10010113/s1, Figure S1: Stacked biovolume (mean ± SD) of *E. huxleyi* (orange) and *C. affinis* (green) at the end of each batch cycle over the course of time; Figure S2: Daily measurements of cell density of *E. huxleyi* (orange) and fluorescence of *C. affinis* (green) at batch cycle three under variable batch cycle length (long batch cycle); Figure S3: Measurements (mean ± SD) of nitrate (red), phosphate (green), and silicate (blue) at the end of batch cycles one (7 days), two (4 days) and three (10 days) under variable batch cycle length in mix-cultures; Figure S4: Measurements of nitrate (red), phosphate (green) and silicate (blue) in mix-cultures at batch cycle seven from day four to seven; Table S1: Primer sequences for microsatellite analysis.
