# Supplementary material for: Maintenance of Intraspecific Diversity in Response to Species Competition and Nutrient Fluctuations
Source: Microorganisms. 2022 Jan 6;10(1):113. doi: 10.3390/microorganisms10010113 (PMC8779635; doi:10.3390/microorganisms10010113)
Supplement: Supplementary file 1 [file microorganisms-10-00113-s001.zip › microorganisms-1487644-supplementary.pdf]

Supplement material: Species sorting and growth curves

Total biovolume increased in both mono-and mix-cultures with nitrate concentration (Figure S1). Across all 13 batch cycles biovolume of *C. affinis* increased from 10N:1P to 30N:1P by 205 % and 206 % in mono-cultures and mix-cultures, respectively. While the biovolume of *E. huxleyi* also steadily increased from 10N:1P to 30N:1P by 50 % in mono-cultures, their biovolume in mix-cultures increased from 10N:1P compared to 20N:1P by 56 %, but decreased by 84 % from 20N:1P to 30N:1P. In the mix-culture both species stably coexisted. While *E. huxleyi* dominated total biovolume at the 10N:1P nutrient regime, proportions were more equal at 20N:1P and changed to dominance of *C. affinis* at the 30N:1P regime. These dynamics were similar in both levels of the batch cycle length treatment. However, varying time for growth at variable batch cycle length led to more variable biovolumes over time and an increase in SD compared to fixed batch cycle length, in particular in *E. huxleyi*.

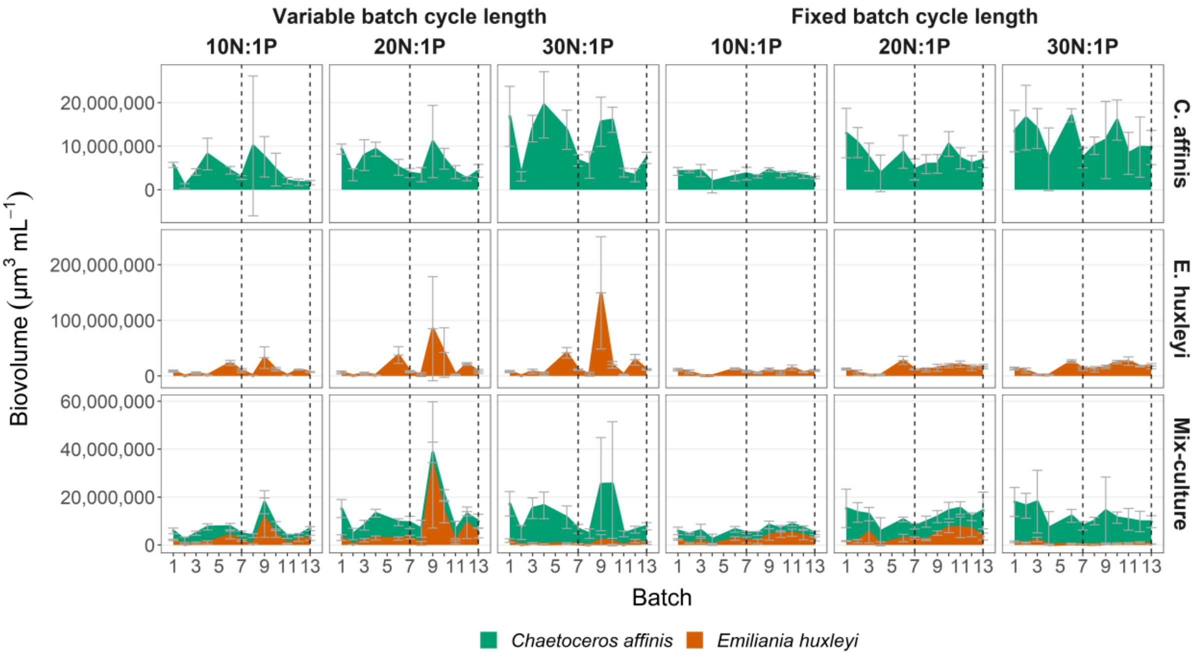

Figure S1. Stacked biovolume (mean±SD) of *E. huxleyi* (orange) and *C. affinis* (green) at the end of each batch cycle over the course of time. Columns show different nutrient regimes (10N:1P, 20N:1P, 30N:1P) at variable and fixed batch cycle length. Rows show mono-cultures of *E. huxleyi* and *C. affinis* and mix-culture. Dashed lines mark the time of genotype composition assessment.

Growth curves based on cell density (*E. huxleyi*) and fluorescence (*C. affinis*) at batch cycle three under variable batch cycle length showed that *C. affinis* reached stationary phase at day

five irrespectively of nutrient regime or culture condition (Figure S2). In turn, E. *huxleyi* continued its growth until the end of the batch cycle without reaching stationary phase.

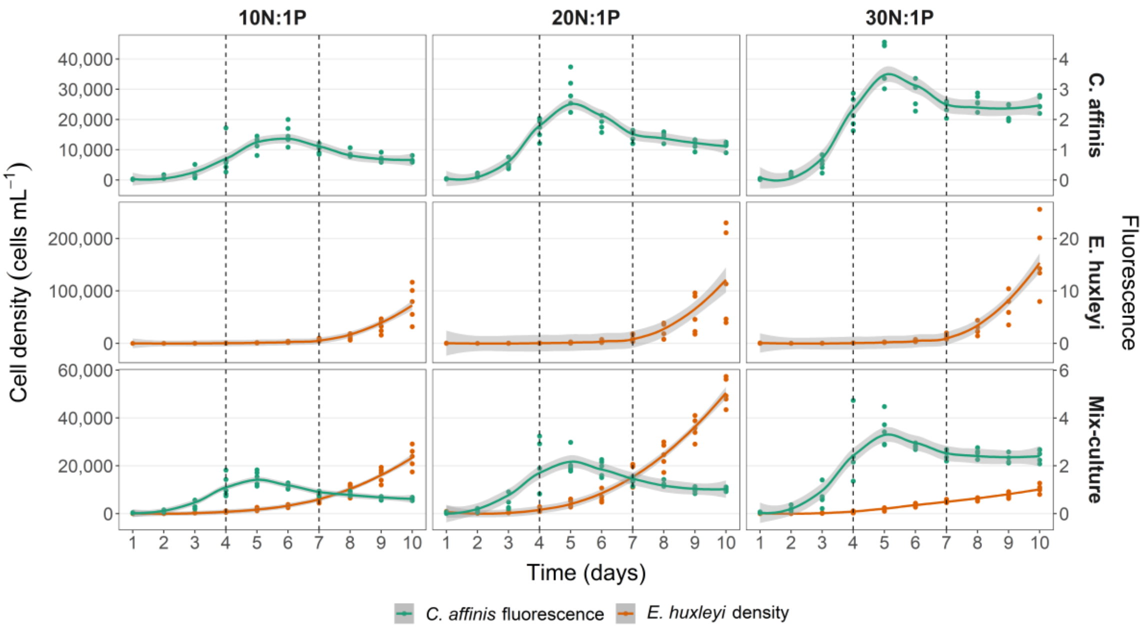

Figure S2. Daily measurements of cell density of *E. huxleyi* (orange) and fluorescence of *C. affinis* (green) at batch cycle three under variable batch cycle length (long batch cycle). Columns show different nutrient regimes (10N:1P, 20N:1P, 30N:1P). Rows show mono-cultures of *E. huxleyi* and *C. affinis* and mix-culture. Dashed lines mark the end of short and normal batch cycles.

Supplement material: Nutrient measurements

Nutrient measurements in the mix-cultures from the end of batch cycles with 4, 7 and 10 days length revealed the different nutrient levels that remained before transfer of cells into a new batch cycle at variable batch cycle length (Figure S3). While after the 4 days batch cycle still nitrate, phosphate and silicate were present in the bottles, in the 7 days batch cycle no nutrients were left except for nitrate in the 30N:1P nutrient regime. After the 10 days batch cycle all nutrients were taken up.

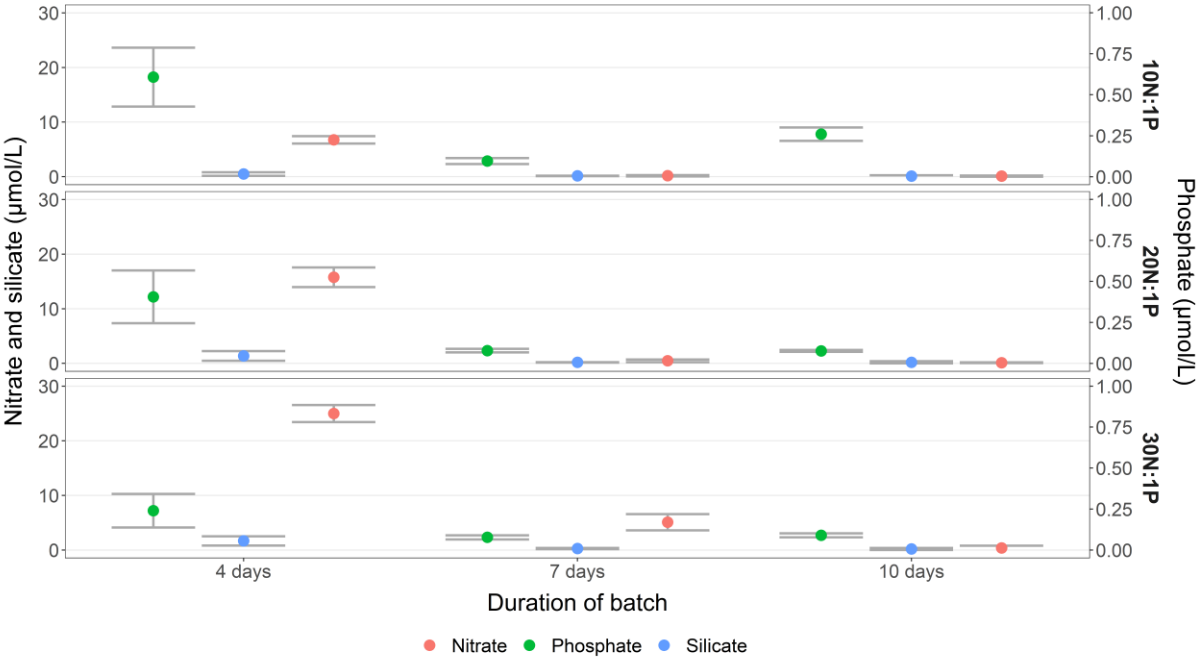

Figure S3. Measurements (mean±SD) of nitrate (red), phosphate (green) and silicate (blue) at the end of batch cycles one (7 days), two (4 days) and three (10 days) under variable batch cycle length in mix-cultures. Columns show different nutrient regimes (10N:1P, 20N:1P, 30N:1P).

Daily nutrient measurements from batch cycle seven in the mix-cultures showed which nutrient was depleted first during the batch cycle (Figure S4). In the 10N:1P nutrient regime nitrate was completely taken up at day 5, although at fixed batch cycle length silicate seemed to be already depleted at day 4, whereas at variable batch it was depleted together with nitrate at day 5. Contrary to the 10N:1P regime, phosphate and silicate were the first nutrients to be depleted at day 5 in both the 20N:1P and 30N:1P nutrient regimes.

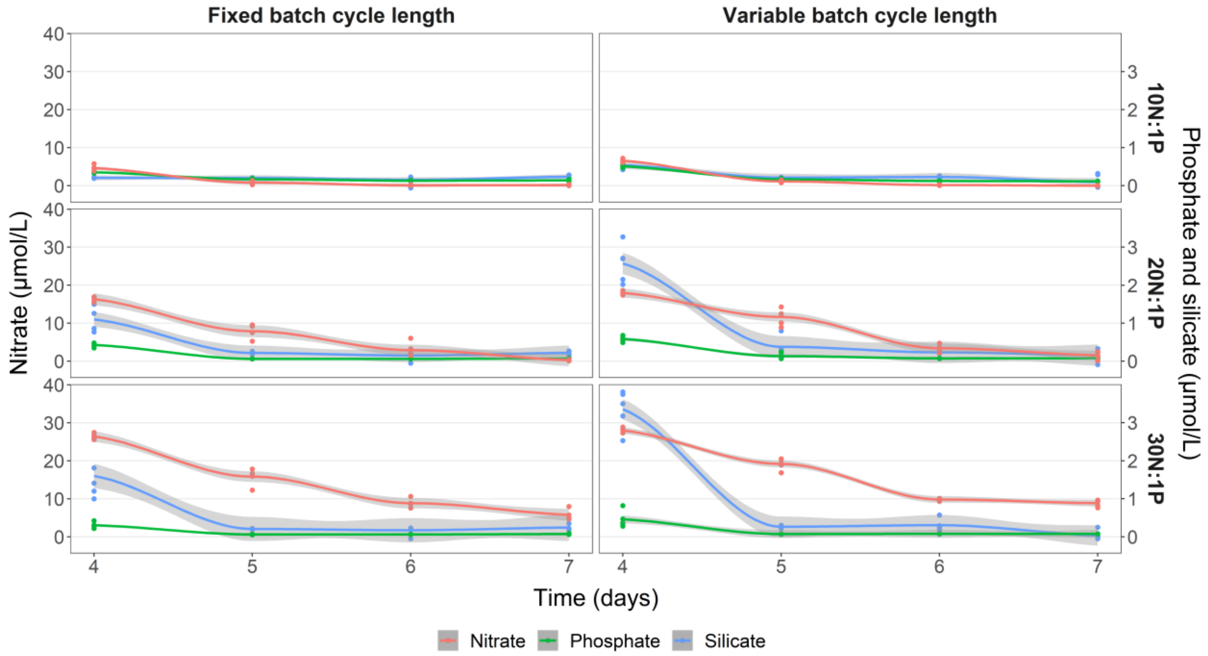

Figure S4. Measurements of nitrate (red), phosphate (green) and silicate (blue) in mix-cultures at batch cycle seven from day four to seven. Columns show batch cycle lengths (fixed and variable) and rows show different nutrient regimes (10N:1P, 20N:1P, 30N:1P).

Supplement material: Genotyping

Assessment of genotype distribution using microsatellites followed methods by Hattich [65] and Listmann [46].

Reisolation

For the mix-cultures, the two species were separated with 20 µm mesh size sieves and then all subsamples were diluted to approximately 3 cells mL^{-1} before transferring about 0.5 mL into each well of a 48 well plate. The well plates were sealed with Parafilm and put in the climate chamber for the cells to grow under experimental conditions. On the following day the *C. affinis* well plates were checked under a microscope and cell chains in every well were counted. Only wells containing one or two chains were marked with the respective quantity until the sum of 10 chains was reached. The well plates were then put back into the climate chamber for the chains to grow. After around 10 days of growth *E. huxleyi* well plates were checked on a black background for white *E. huxleyi* colonies. Wells with one or two colonies were marked with the respective quantity until the sum of 10 colonies was reached. For both species the cells of the marked well plates were then transferred into five 8 mL Sarstedt

tubes, each containing two of the initial isolates. The tubes were filled with f/8 medium [69] and cells were allowed to grow for about two weeks.

DNA extraction, PCR and sequencing

DNA extraction E. huxleyi

From the tubes with settled cells inside, about 5 mL were taken out. The cells in the remaining water were suspended and transferred to 2 mL Eppendorf tubes prior to centrifugation for three minutes at 12000 x g. After removal of the supernatant 20 µL cell pellet was added with 30 µL TE buffer (10 mM Tris HCl and 1 mM EDTA) to a 96 well plate. The well plate was sonicated for three minutes in a water bath and then heated at 58°C for one hour.

DNA extraction C. affinis

About 1 mL of settled cells were taken out of the Sarstedt tube and centrifuged in a 2 mL Eppendorf tube at 12000 x g for three minutes. The supernatant was removed and 20 µL Proteinase K (0.1 mg mL^{-1}) and 500 µL SDS (5 mg mL^{-1}) were added and then kept for one hour at 55°C. After addition of 80 µL 5M NaCl and 150 µL CTAB buffer (2% Hexadecyltrimethyl ammonium bromide, 1% Polyvinylpyrrolidone, 100 mM Tris HCl, 20 mM EDTA, 1.4 M NaCl) samples were kept at 65°C for another hour. Then 700 µL 24:1 chloroform isoamyl alcohol was added and samples were mixed. After centrifugation at 2000 x g for five minutes 500 µL of the aqueous supernatant was transferred into 1.5 mL Eppendorf tubes and mixed gently with 300 µL isopropanol. Samples were kept at room temperature overnight and then centrifuged at 12000 x g for 15 minutes. After removal of the supernatant the remaining pellet was washed with 300 µL 70% ethanol and centrifuged at 12000 x g for three minutes. The washing step was repeated three times before the pellet was air dried and then suspended with 50 µL TE buffer. Afterwards, extracted DNA samples were frozen at -20°C.

Polymerase chain reaction (PCR)

The microsatellite amplification was done by using 1 µL DNA Extract with 5 µL Multiplex Mix (Qiagen), 1 µL H2O, 2 µL Q-Solution (Qiagen) and 2 × 0.25 µL 5pM forward and 2 × 0.25 µL 5pM reverse primers. Primers P02F11 and P02E10 were used for *E. huxleyi* and C.a_LL_8 and C.a_LL_17 were used for *C. affinis* (Table 2). The forward primers were labelled with

HEX or FAM for identification when sequenced. The reagents were filled in a 96-well plate and put in thermal cyclers PTC-200 (MJ Research) and FlexCycler (Analytik Jena). The program was set to 15 minutes initial denaturing at 95°C, followed by 28 cycles of denaturing for 30 seconds at 94°C, annealing for 90 seconds at 57°C and 60 seconds extension at 72°C. The final extension was set to 30 minutes at 60°C before indefinite hold at 4°C.

Table S1. Primer sequences for microsatellite analysis.

Primer name	Forward sequence (5′ → 3′) [Label]	Reverse sequence (5′ → 3′)
P02F11	CTCGTGTGGCTATGCCTATG [FAM]	TCCAAGAGCAAAGTGCAAAA
P02E10	CTCGTGTAGTCGGGGAGTGT [HEX]	CACGCGGTCCAATATTACCT
C.a_LL_8	GACGCTGGTAGTTTCGTTTG [HEX]	AGTCCCTTGGAAATGGACTG
C.a_LL_17	GGGGTAATGAAATCTTTGGTGC [FAM]	GTACTGATTATCAACAGGTGCTC

Sequencing

For every reaction a mix of 8.75 µL HiDi (Qiagen) and 0.25 µL GeneScan 350 ROX (Applied Biosystems) was filled with 1 µL PCR product in a 96-well plate. After denaturation for 2 minutes at 95 °C samples were sequenced using 3130xl Genetic Analyzer (Applied Biosystems).
